# Identification of stably expressed Internal Control Genes (ICGs) for normalization of expression data in liver of C57BL/6 mice injected with beta casomorphins

**DOI:** 10.1371/journal.pone.0282994

**Published:** 2023-05-05

**Authors:** Anurag Kumar, Monika Sodhi, Manishi Mukesh, Amandeep Kaur, Gaurav Bhakri, Vipul Chaudhary, Preeti Swami, Vishal Sharma, Ashok Kumar Mohanty, Ranjit S. Kataria

**Affiliations:** 1 Animal Biotechnology Centre, National Dairy Research Institute, Karnal, Haryana, India; 2 Animal Biotechnology Division, National Bureau of Animal Genetic Resources, Karnal, Haryana, India; University of Illinois, UNITED STATES

## Abstract

In recent years, beta-casomorphin peptides (BCM7/BCM9) derived from the digestion of cow milk have drawn a lot of attention world over because of their proposed impact on human health. In order to evaluate the transcriptional modulation of target genes through RT-qPCR in response to these peptides, availability of appropriate reference or internal control genes (ICGs) will be the key. The present study was planned to identify a panel of stable ICGs in the liver tissue of C57BL/6 mice injected with BCM7/BCM9 cow milk peptides for 3 weeks. A total of ten candidate genes were evaluated as potential ICGs by assessing their expression stability using software suites; geNorm, NormFinder and BestKeeper. The suitability of the identified ICGs was validated by assessing the relative expression levels of target genes, *HP* and *Cu/Zn SOD*. Based on geNorm, *PPIA* and *SDHA* gene pair was identified to be most stably expressed in liver tissue during the animal trials. Similarly, NormFinder analysis also identified *PPIA* as the most stable gene. BestKeeper analysis showed crossing point SD value for all the genes in the acceptable range that is closer to 1. Overall, the study identified a panel of stable ICGs for reliable normalization of target genes expression data in mice liver tissues during BCM7/9 peptides trial.

## Introduction

In recent years, beta-casomorphin (BCM) peptides from cow milk have attracted lot of attention due to their potential health implications [1]. These peptides are produced after the proteolytic digestion of beta casein present in milk [2]. Amongst all the BCMs, BCM7 and BCM9 peptides, produced from A1 and A2 beta casein type variants respectively, are most widely studied [1, 3]. Till now, 15 variants of beta casein gene have been reported and among these A1 and A2 type variants occur in varying proportions in different cattle breeds [4–6]. A1 variant is characterized by the presence of histidine at 67 amino acid position, while in A2 variant histidine is replaced with proline. After proteolytic digestion, this non-synonymous change results in the generation of two different casomorphins; BCM7 in A1 and BCM9 in A2 type beta casein [7]. Various studies have correlated BCM7 peptide generated during proteolytic digestion of A1 type milk with many diseases like diabetes, cardiovascular disease, neurological disorders, pulmonary ailment, digestive disorders and sudden infant death syndrome [8–16]. On the contrary, BCM9 generated from proteolytic digestion of A2 type beta-casein has not been associated with any of such ill-health effects [8, 9].

Worldwide, cattle (*Bos taurus* or *Bos indicus*) is the prominent livestock species for the dairy sector. The distribution of A1 and A2 types of β-casein variants in two cattle types (taurine or indicus) is highly skewed. The taurine cattle exhibit high frequency of A1 type variants while indicus cattle have a high proportion of A2 type variants [4, 17]. Such scenario has prompted various scientific groups to document the A1/A2 profile in breeds across different countries [5, 6, 18, 19]. India is home to large number of native cattle breeds of *Bos indicus* lineage, possessing a very high proportion or complete fixation of A2 type allele [4, 19]. Due to such kind of allelic status of beta casein, Indian cattle are generally referred as a natural resource of A2 milk. However, crossbreeding with exotic/taurine cattle to increase milk production of indigenous breeds also introduced A1 type allelic variants among the crossbred cattle. The plausible association of A1 milk/BCM7 with different diseases makes it pertinent to validate the effects of A1 or A2 milk variants on human health using well designed experimental plan. In the past few years, various animal trials have been conducted to establish potential adverse outcomes of BCM7 with contradictory results in different species including mice [8, 9], rat [20–22], rabbits [23], human [24].

Gene expression changes during any such experimental trial involving bioactive peptides have always been used as an important parameter to evaluate the pathophysiological alterations in the target tissue. In order to quantify the transcript changes accurately in different treatment groups, availability of a panel of appropriate internal control gene(s) is often considered an important prerequisite. Unfortunately, many studies have utilized Beta-actin and *GAPDH* as reference genes to normalize target gene data, without validating their stability across control and treatment groups. Nowadays, much emphasis is being given on identifying suitable internal control genes for a particular experimental setup [25, 26]. This approach has been successfully utilized by several investigators and specific panel of ICGs in different tissues, cells, and species for different experimental conditions have been identified [27–30].

Keeping this in mind, the present study was conducted to identify an appropriate panel of ICGs to accurately normalize the target gene expression data generated on liver tissue of C57BL/6 mice, in response to BCM7/9 peptide treatment.

## Materials and methods

### Animal handling and sampling

For the present study, 7–8 week old male C57BL/6 mice (n = 35) were housed in a room maintained at 21±2°C, 60–70% RH, and normal day/night cycle. All the mice were given commercial chow diet and free access to drinking water. The animals were allowed to acclimatize to the room conditions for at least one week before dividing them randomly into five groups (7 mice/per group). However, the final data was analyzed across 30 animals survived during the experiment [BCM7-200 (n = 7), BCM7-400 (n = 5), BCM9-200 (n = 6), BCM9-400 (n = 6) and control (n = 6)]. The control group received physiological saline while the other groups received either 200 μg or 400 μg of BCM7 or BCM9 synthetic peptides (ComFax Systems India, Chandigarh, India) through the intraperitoneal route. The doses of 200 and 400 μg for both the peptides were decided on the basis of preliminary trial conducted to standardize dose based on rise in blood glucose levels following peptide treatment. The animals were injected with BCM7 and BCM9 peptides daily once for a period of three weeks and regularly monitored for their health. The ethical approval for this study was obtained from the Institutional Animal Ethics Committee (IAEC) of ICAR-National Dairy Research Institute, Karnal, India (Reg. No. 1705/GO/Re/SL/13/CPCSEA), registered under the committee for the purpose of control and supervision of experiments on animals (CPCSEA). All the guidelines of IAEC for handling the animals were strictly followed. At the end of the experiment, animals were given gaseous anesthesia (diethyl ether), sacrificed by decapitation, liver tissues were removed and stored immediately in RNALater solution (Sigma Aldrich, USA). The liver tissue samples were brought to the laboratory and stored at -20°C until further processing.

### RNA isolation, cDNA synthesis and primers

Total RNA was isolated using Trizol reagent (Invitrogen, Corp., CA) following the manufacturer’s protocol. Approximately 100 mg of tissue was homogenized using a handgrip homogenizer (T 10 basic ultra-turrax^R^, IKA, Germany). The extracted RNA was purified to remove the traces of genomic DNA using RNeasy Mini kit and on column digestion with RNase-free DNase enzyme (Qiagen, Germany). Concentration and purity of RNA was checked using NanoDrop ND-1000 spectrophotometer (NanoDrop Technologies). Absorbance ratio 260/280 and 260/230 were calculated to assess the purity of isolated RNA. For cDNA synthesis, 2μg of purified RNA was reverse transcribed using RevertAid First Strand cDNA Synthesis Kit (Thermo Fisher Scientific, USA). A panel of 10 potential reference genes representing different functional categories and reported in different studies was shortlisted. The primers sequences were either taken from the literature or designed using Primer 3.0 software (version 0.4.0) (http://bioinfo.ut.ee/primer3-0.4.0/) based on mice sequences available in the Ensembl Genome Browser (https://asia.ensembl.org/index.html). Details of the genes, primers’ sequences, product size, etc. are summarized in Table 1.

**Table 1:** Gene name, symbol, GenBank accession numbers, primer sequences and amplicon length for each evaluated ICG and target gene.

Genes	Ensemble Sequence ID//Reference	Forward Primer	Reverse Primer	Product Size (bp)
*ACTB* (Beta-actin)	ENSMUST00000100497.10	5’CTCTTTTCCAGCCTTCCTTC3’	5’GGTCTTTACGGATGTCAACG3’	99
*B2M*(Beta-2-Microglobulin)	ENSMUST00000102476.4	5'CTGGTCTTTCTGGTGCTTGTC3'	5'GTATGTTCGGCTTCCCATTC3'	109
*GUSB* (Glucoronidase Beta)	ENSMUST00000026613.13	5'ACCAGCCACTATCCCTACTCA3'	5'GCCACAGACCACATCACAA3'	200
*HMBS* (Hydroxymethyl bilane Synthase)	ENSMUST00000077353.14	5’GGATGTGCCTACCATACTACCTC3'	5'GGTTTCCAGGGTCTTTCCA3'	115
*HPRT* (Hyoxanthine Phosphoribosyl Transferase)	ENSMUST00000026723.8	5'AGTTCTTTGCTGACCTGCTG3'	5'TTATGTCCCCCGTTGACTG3'	126
*PGK1* (Phoshoglycerate Kinase 1)	ENSMUST00000081593.12	5'GAGGAAGAAGGGAAGGGAAA3'	5'GCAGATTCACACCCACCAT3'	166
*PPIA* (Peptidyl prolyl Isomerase A)	ENSMUST00000132846.1	5'CAGGGTGGTGACTTTACACG3'	5'ATGGACAAGATGCCAGGAC3'	113
*SDHA* (Succinate Dehydrogenase Complex flavoprotein subunit A)	ENSMUST00000022062.7	5'GCCAGGAACACTCCAAAAAC3'	5'GCCTACAACCACAGCATCAA3'	139
*TBP* (Tata—Binding Protein)	ENSMUST00000162505.7	5'TATCACTCCTGCCACACCA3'	5'CAGCCAAGATTCACGGTAGA3'	84
*TFR2* (Transferrin Receptor 2)	ENSMUST00000031729.12	5'CCCAGTTCCCTCCAGTAGAA-3'	5'GTTGACCACAAGGCGTAAGTC3'	185
*HP* (Haptoglobin)	ENSMUST00000074898.8	5’GCTATGTGGAGCACTTGGTTC3’	5’CACCCATTGCTTCTCGTCGTT3’	91
*Cu/Zn SOD* (Copper/Zinc Superoxide Dismutase)	[31]	5’AAGGCCGTGTGCGTGCTGAA3’	5’CAGGTCTCCAACATGCCTCT3’	246

### Quantitative PCR (qPCR)

Each primer pair was tested for its PCR amplification specificity before subjecting to qPCR analysis. Normal PCR amplification was carried out in a reaction volume of 10 μl containing 1 μL of 10×PCR buffer, 10 mM dNTPs mix, 10 μM each of forward and reverse primers, 1 μL of cDNA and 0.2 μL of Taq DNA polymerase (New England Biolabs). After confirming the specific amplification of each transcript on agarose gel, real-time quantitative PCR (qPCR) was performed using LightCycler 480 system (Roche Life Sciences, Germany) in a 96-well white plate (Brand GmBH, Germany). Each reaction was performed in a final volume of 10 μl containing 2 μl of cDNA (30 times diluted cDNA, synthesized from 2 μg of RNA) 0.3 μl each of forward and reverse primer (10 pmol/μl), 2.4 μl nuclease free water and 5 μl 2X Kapa SYBRGreen master mix (Sigma-Aldrich). For each gene, 6 biological replicates and 2 technical replicates were analyzed along with non-template controls (NTC). For estimating the PCR reaction efficiency, a six-point standard curve was run for each primer pair. The qPCR expression data for each gene was extracted in the form of crossing points or quantification cycle (Cq). The data was acquired using the ‘second derivative maximum’ method, as computed by LightCycler Software 3.5 (Roche Diagnostics) and subjected to analysis. Care was taken to follow the “Minimum information for publication of Quantitative Real-time PCR Experiments” (MIQE) guidelines while designing the real-time PCR based gene expression experiment [39].

### Analysis of data for gene expression stability

In this study, three different statistical tools: geNorm, based on geometric averaging of multiple ICGs; Normfinder, using model-based variance estimation approach and BestKeeper measuring pair-wise correlations [32–34] were used to assess the expression stability of ten potential reference genes. The Cq value of each gene was first converted into relative quantities using 2^−ΔCq^ formula where ΔCq value is obtained by subtracting minimum Cq value from each corresponding Cq value. The mean value of relative quantities of each biological replicate was used as input data for geNorm and NormFinder analysis. The geNorm software (http://medgen.ugent.be/∼jvdesomp/geNorm/) estimates the expression stability (M) of individual gene and ranks the genes according to M value [32]. The lower M value indicates higher expression stability. M-value calculation is based on average pairwise variation of a particular gene in comparison with all other genes. The geNorm software also calculates pairwise variation (V value), which helps to identify the optimal number of ICGs to be used for normalization of expression data. Briefly, contribution of each gene to the variance of normalization factor ratio was calculated to illustrate the effect of adding or removing a particular gene from the final set of ICGs. A threshold V value of 0.15 or less is generally considered as acceptable. NormFinder software (http://www.mdl.dk/) was another tool utilized to assess the expression stability of the ICGs [33]. NormFinder algorithm ranks the candidate ICGs by combining the intra and inter-group variations of expression in a sample set. Gene(s) with the lowest values are considered as the most stably expressed gene(s) in the experimental sample sets. Another statistical tool employed in the study was BestKeeper. This software uses the raw Cq values as input and there is no requirement of converting Cq values into relative quantities. The analysis is based on the assumption that stably expressed genes will be highly correlated to each other. This tool helps to calculate crossing point standard deviations of Cq values (SD, ±CP), coefficient of variance (CV) and Pearson’s correlation coefficient for each reference gene [34].

### Validation of internal control genes

To verify the stability of proposed internal control genes (ICGs), expression analysis of target genes, cytosol copper superoxide dismutase (*Cu/Zn SOD*) and acute phase anti-inflammatory protein coding haptoglobin (*HP*) reported to be associated with diabetes in mice liver, was performed. Combination of two most stable and two least stable ICGs identified in the present study; and *Beta-actin* (*ACTB*)—one of the commonly used ICG were used to normalize the expression data across treatment and control groups. Relative expression profiles of target genes across different control and treatment groups were evaluated to assess if normalization with different ICGs affect the reliability and accuracy of expression analysis.

## Results

### Efficiency and specificity of PCR

**Fig 1:**
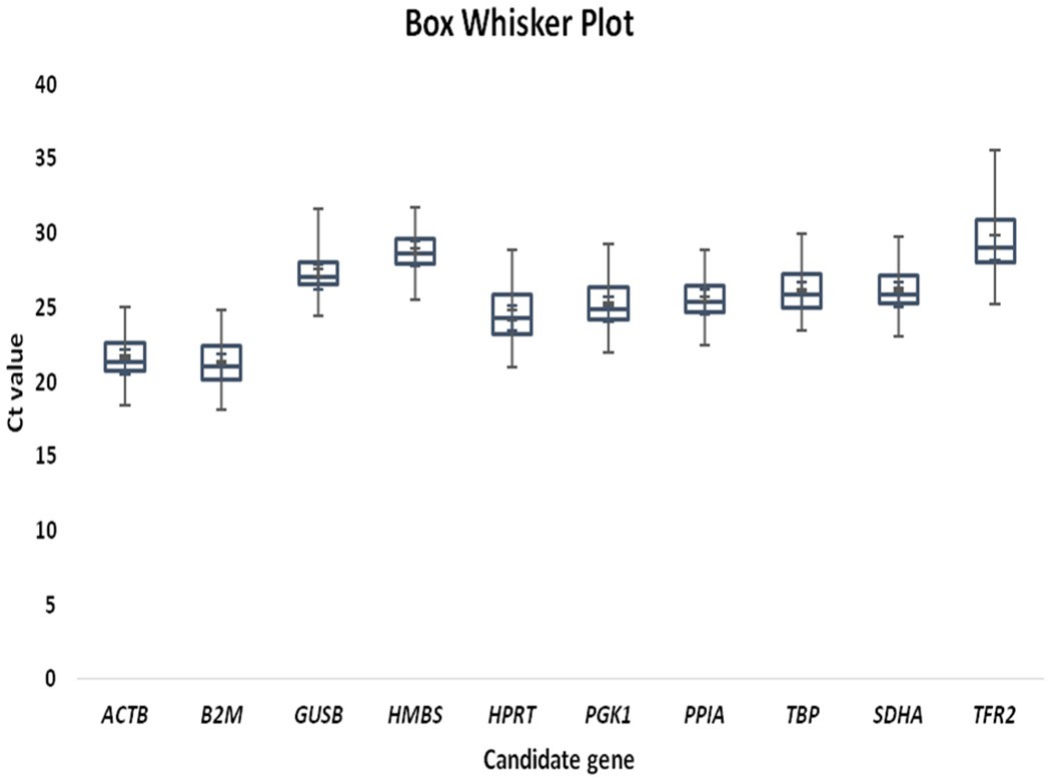
The data of expression levels of individual candidate ICGs staged as quantification cycle (Cq) values of each gene in the box-whisker diagram. The median is shown as a line across the box while whiskers indicate maximum and minimum values.

In the present study, a total of 10 genes were evaluated for their expression stability across 30 liver tissue samples of C57BL/6 mice injected with BCM7 and BCM9 peptides for 3 weeks. The RNA extracted from each tissue sample was of good quality as indicated by A260/A280 and 260/230 optical density ratios of 2.05 ± 0.04 (range: 1.94–2.09) and 2.12 ± 0.05 (range: 2.0–2.21) respectively (S1 Table). Average yield of RNA obtained was 3103.62 ± 250.36 ng/mg of liver tissue. The specificity of each primer pair was confirmed by the presence of distinct amplified product of expected size (S1 Fig). The melt curve analysis also suggested specific amplification as no primer-dimer formation was observed for any of the genes (S2 Fig). The PCR amplification efficiency, R^2^ and slope for individual reference gene are summarized in Table 2. Among the 30 animals studied, no outlier was detected and all the Cq values were used for the analysis. The Cq values of individual genes were used to visualize their expression pattern across different groups (Fig 1). Three software that are commonly used in such study namely; geNorm, NormFinder and BestKeeper, were utilized to determine the expression stability of all candidate ICGs.

**Table 2:** Slope of standard curve, coefficient of correlation and percentage efficiency of PCR reaction for each ICG.

Genes	Slope	R^2^	Efficiency (%)
*ACTB*	-3.144	0.999	108.001
*B2M*	-3.278	0.991	101.879
*HMBS*	-3.099	0.972	110.23
*GUSB*	-2.823	0.987	126.052
*HPRT*	-3.09	0.991	110.696
*PGK1*	-3.234	0.980	103.827
*PPIA*	-3.36	0.985	98.452
*TBP*	-2.931	0.994	119.372
*SDHA*	-3.389	0.991	97.287
*TFR2*	-3.748	0.967	84.846

### Ranking of genes based on geNorm analysis

**Fig 2:**
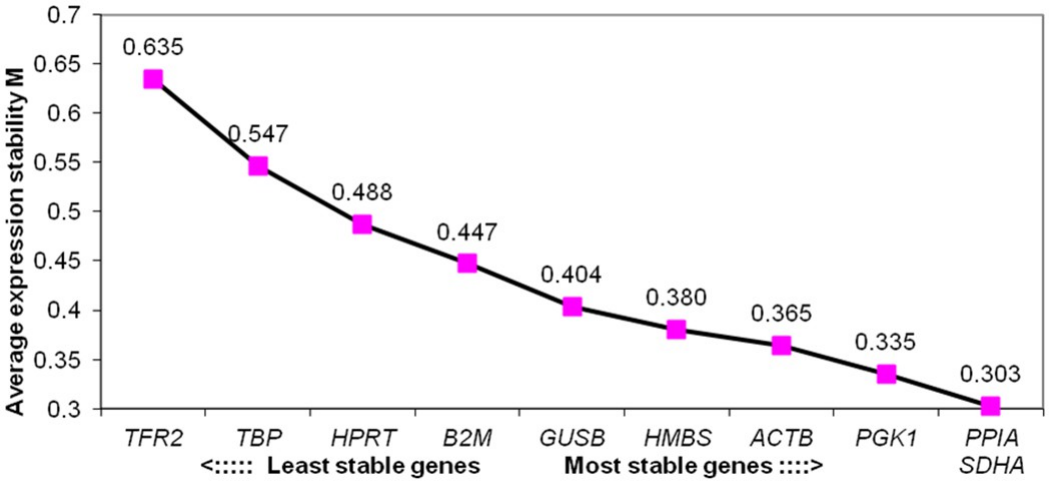
ICGs ranking based on average expression stability values (M values) using geNorm software.

**Fig 3:**
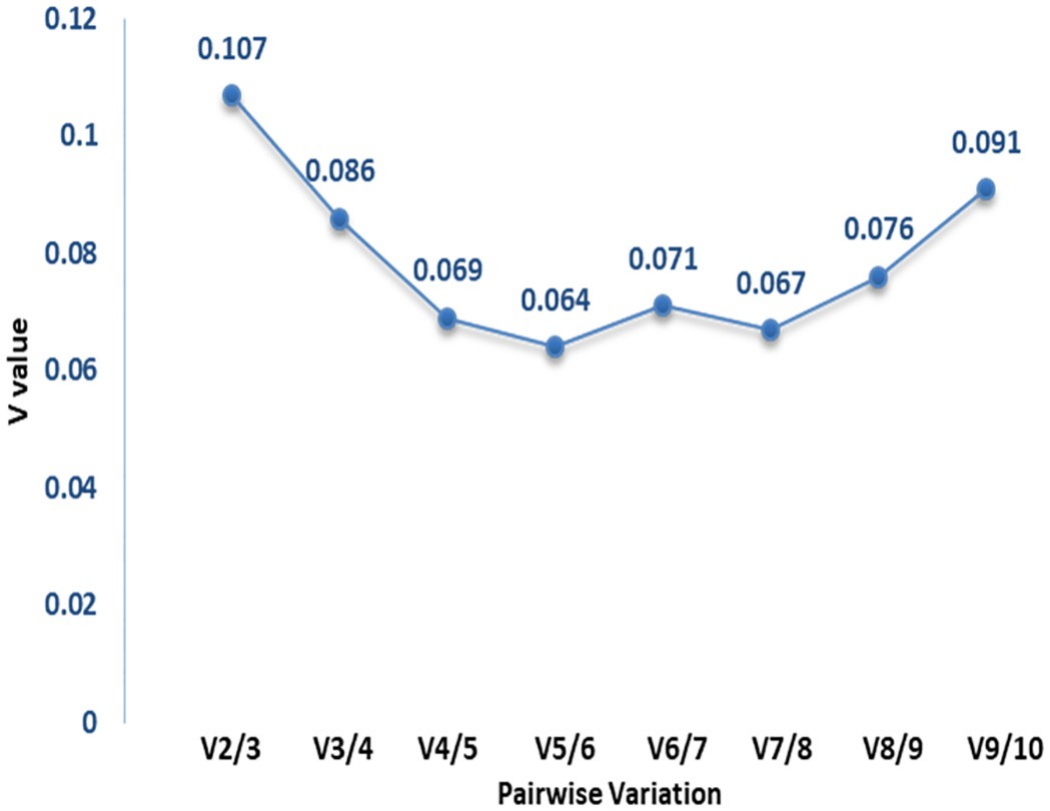
Pairwise variation in ICGs by calculating V value through geNorm for determining the optimal number of genes for normalization of target genes.

The geNorm based ranking of reference genes using their average expression stabilities or M values were within an acceptable range (<1.5), indicating their expression stability across five biological groups (Table 3, Fig 2). Amongst 10 genes evaluated, minimum M value (0.303) was observed for *PPIA* and *SDHA* genes. The M value is inversely correlated to gene expression stability; hence *PPIA* and *SDHA* genes were ranked as most stable reference genes by the software. On the other hand, the highest M values were observed for *TBP* (0.547) and *TFR2* (0.635) genes, indicating that *TBP* and *TFR2* having the most variable expression across groups and therefore these two were ranked as least stable. Overall geNorm ranking of genes based on expression stability and stepwise exclusion criteria were; *PPIA = SDHA>PGK1>ACTB>HMBS>GUSB>B2M>HPRT>TBP>TFR2*. Additionally, the pairwise variation analysis (Vn/Vn + 1) using geNorm was performed to identify optimal number of genes to be used for accurate normalization of qPCR data. In pairwise variation analysis, a cutoff value of 0.15 is generally recommended [32]. In the present analysis, maximum V value observed for V2/3 pair was 0.107, which is less than cut off limit of 0.15 (Fig 3). This suggests that the addition of any other reference gene will make no significant difference in improving the normalization accuracy. Based on the analysis, it can be suggested that for the particular experimental set up as described in the present study, only two genes; *PPIA* and *SDHA* will act as appropriate ICGs.

**Table 3:** Ranking of candidate gene expression based on M value using geNorm algorithm.

Genes	M Value (<1.5) (geNorm)	Rank
*PPIA*	0.303	1
*SDHA*	0.303	1
*PGK1*	0.335	2
*ACTB*	0.365	3
*HMBS*	0.380	4
*GUSB*	0.404	5
*B2M*	0.447	6
*HPRT*	0.488	7
*TBP*	0.547	8
*TFR2*	0.635	9

### Ranking of genes based on NormFinder analysis

**Fig 4:**
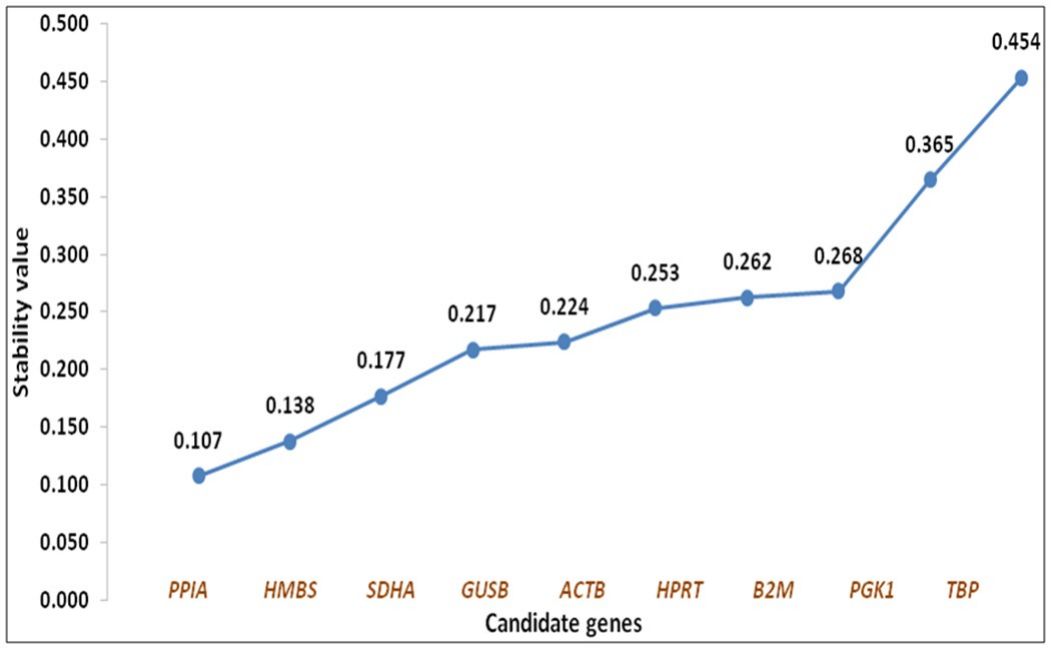
Scatter plot of stability values for each candidate ICGs calculated by NormFinder, representing the most stable and least stable gene.

**Fig 5:**
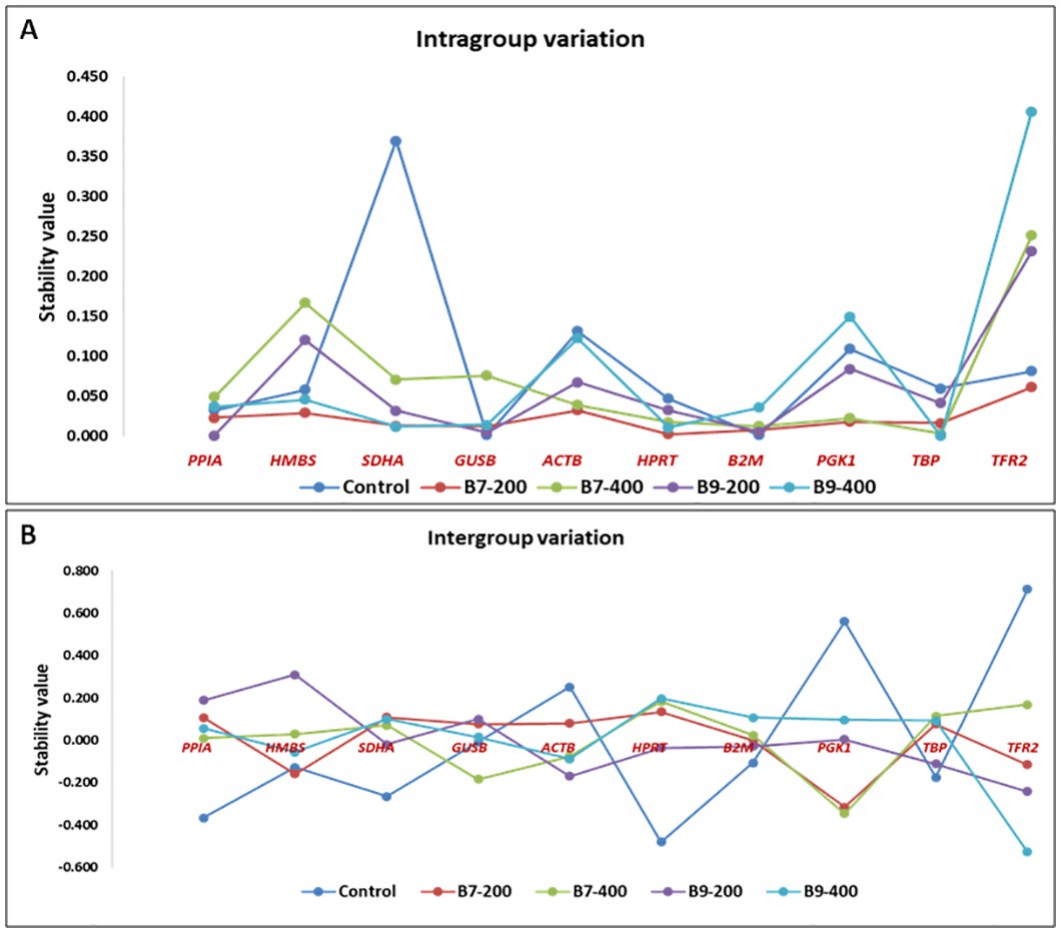
Scatter plot of stability value observed using NormFinder. (A) Intra group variation of all 10 candidate ICGs in each group. (B) Inter group variation of each candidate ICGs in different groups.

Ranking of genes based on NormFinder analysis revealed *PPIA* (0.107) to be the most stable followed by *HMBS* (0.138) and *SDHA* (0.177) (Fig 4), while *TFR2* gene was observed to be the least stable gene with a stability value of 0.454 (Table 4). The overall ranking of genes as per NormFinder analysis was; *PPIA>HMBS>>SDHA>GUSB>ACTB>HPRT>B2M>PGK1>TBP>TFR2* (Table 4) and combination of *PPIA* and HMBS genes, was found to be most stably expressed ones. Set of genes with most stable (*PPIA*) and least stable (*TFR2*) expression were similar as revealed by geNorm and NormFinder analysis. NormFinder was also used to estimate inter and intragroup variations. The inter group variation across all the treatment groups showed *PPIA*, *HMBS* and *SDHA* genes to be more stable among the group of genes analyzed (Fig 5).

**Table 4:** Ranking of ICGs across all the groups according to their expression stability by NormFinder software.

Genes	Stability Value (NormFinder)	Rank
*PPIA*	0.107	1
*HMBS*	0.138	2
*SDHA*	0.177	3
*GUSB*	0.217	4
*ACTB*	0.224	5
*HPRT*	0.253	6
*B2M*	0.262	7
*PGK1*	0.268	8
*TBP*	0.365	9
*TFR2*	0.454	10

### Ranking of genes based on BestKeeper analysis

BestKeeper algorithm ranks the stability of candidate internal control genes based on standard deviation (SD) of their Cq values. In BestKeeper analysis, *TBP* gene showed least SD value (0.81) while *TFR2* had the highest SD value (1.32) (Table 5). Out of the 10 genes, the crossing point SD values were > 1.0 for six genes (*SDHA*, *GUSB*, *HPRT*, *PGK1*, *SDHA* and *TFR2*). BestKeeper provides the inter-gene relationship for the candidate internal control genes gene pairs on the basis of Pearson correlation coefficients (r). Pearson correlation coefficients for all the gene pairs were observed closer to 1.0 (Table 6). BestKeeper also estimates the relationship between internal control genes (ICGs), in terms of BestKeeper vs. candidate gene correlation coefficient (r), closer to 1.0 and the p value for all the genes. The highest correlation between BestKeeper and ICGs was witnessed for *PPIA* (r = 0.989) and least for *TBP* (r = 0.887) (Table 6).

**Table 5:** Expression stability analysis of all 10 candidate reference genes (ICGs) by BestKeeper software.

Cq data of reference Genes:										
	*ACTB*	*B2M*	*GUSB*	*HMBS*	*HPRT*	*PGK1*	*PPIA*	*TBP*	*SDHA*	*TFR2*
N	30	30	30	30	30	30	30	30	30	30
geo Mean [Cq]	21.27	20.93	27.21	28.46	24.20	24.93	25.31	25.81	25.98	29.19
ar Mean [Cq]	21.31	20.96	27.25	28.49	24.24	24.97	25.34	25.83	26.02	29.24
min [Cq]	18.97	18.98	24.89	26.16	22.09	22.71	23.13	24.29	23.66	26.21
max [Cq]	23.77	23.41	30.59	30.72	27.32	27.79	27.77	28.52	28.49	33.77
std dev [± Cq]	1.00	0.85	1.08	0.94	1.06	1.11	0.94	0.81	1.07	1.32
CV [% Cq]	4.70	4.05	3.95	3.32	4.37	4.45	3.73	3.13	4.10	4.51
min [x-fold]	-4.93	-3.85	-4.98	-4.93	-4.30	-4.66	-4.53	-2.87	-5.00	-7.84
max [x-fold]	5.68	5.57	10.40	4.79	8.69	7.26	5.49	6.55	5.69	24.02
Std dev [± x-fold]	2.00	1.80	2.11	1.92	2.09	2.16	1.92	1.75	2.09	2.49

N = number of samples, geo Mean[Cq] = geometric mean of Cq; ar Mean[Cq] = arithmetic mean of Cq; min [Cq] and max [Cq] = extreme values of Cq; Std dev [±Cq] = standard deviation of the Cq; CV [%Cq] = coefficient of variation expressed as a percentage on the Cq values; min [x-fold] and max [x-fold] = extreme values of expression levels expressed as absolute x-fold over or under coefficient; std dev[±x-fold] = standard deviation of the absolute regulation coefficients.

**Table 6:** Pairwise comparison using Pearson correlation coefficient (r) and BestKeeper vs reference genes index using BestKeeper.

Pearson correlation coefficient (r)										
vs.	*ACTB*	*B2M*	*GUSB*	*HMBS*	*HPRT*	*PGK1*	*PPIA*	*TBP*	*SDHA*	*TFR2*
*B2M*	0.920	-	-	-	-	-	-	-	-	**-**
p-value	0.001	-	-	-	-	-	-	-	-	**-**
*GUSB*	0.935	0.890	-	-	-	-	-	-	-	**-**
p-value	0.001	0.001	-	-	-	-	-	-	-	**-**
*HMBS*	0.952	0.930	0.931	-	-	-	-	-	-	**-**
p-value	0.001	0.001	0.001	-	-	-	-	-	-	**-**
*HPRT*	0.886	0.899	0.856	0.940	-	-	-	-	-	**-**
p-value	0.001	0.000	0.001	0.001	-	-	-	-	-	**-**
*PGK1*	0.964	0.913	0.970	0.933	0.876	-	-	-	-	**-**
p-value	0.001	0.001	0.001	0.001	0.001	-	-	-	-	**-**
*PPIA*	0.964	0.945	0.940	0.970	0.935	0.964	-	-	-	**-**
p-value	0.001	0.001	0.001	0.001	0.001	0.001	-	-	-	**-**
*TBP*	0.800	0.790	0.777	0.887	0.894	0.751	0.859	-	-	**-**
p-value	0.001	0.001	0.001	0.001	0.001	0.001	0.001	-	-	**-**
*SDHA*	0.949	0.930	0.968	0.965	0.926	0.977	0.981	0.838	-	**-**
p-value	0.001	0.001	0.001	0.001	0.001	0.001	0.001	0.001	-	**-**
*TFR2*	0.806	0.833	0.815	0.873	0.896	0.773	0.867	0.899	0.849	**-**
p-value	0.001	0.001	0.001	0.001	0.001	0.001	0.001	0.001	0.001	**-**
**BestKeeper vs**.										
Coeff. of corr. [r]	0.964	0.950	0.954	0.983	0.955	0.959	0.989	0.887	0.985	0.903
p-value	0.001	0.001	0.001	0.001	0.001	0.001	0.001	0.001	0.001	0.001

### Validation of internal control gene

**Fig 6:**
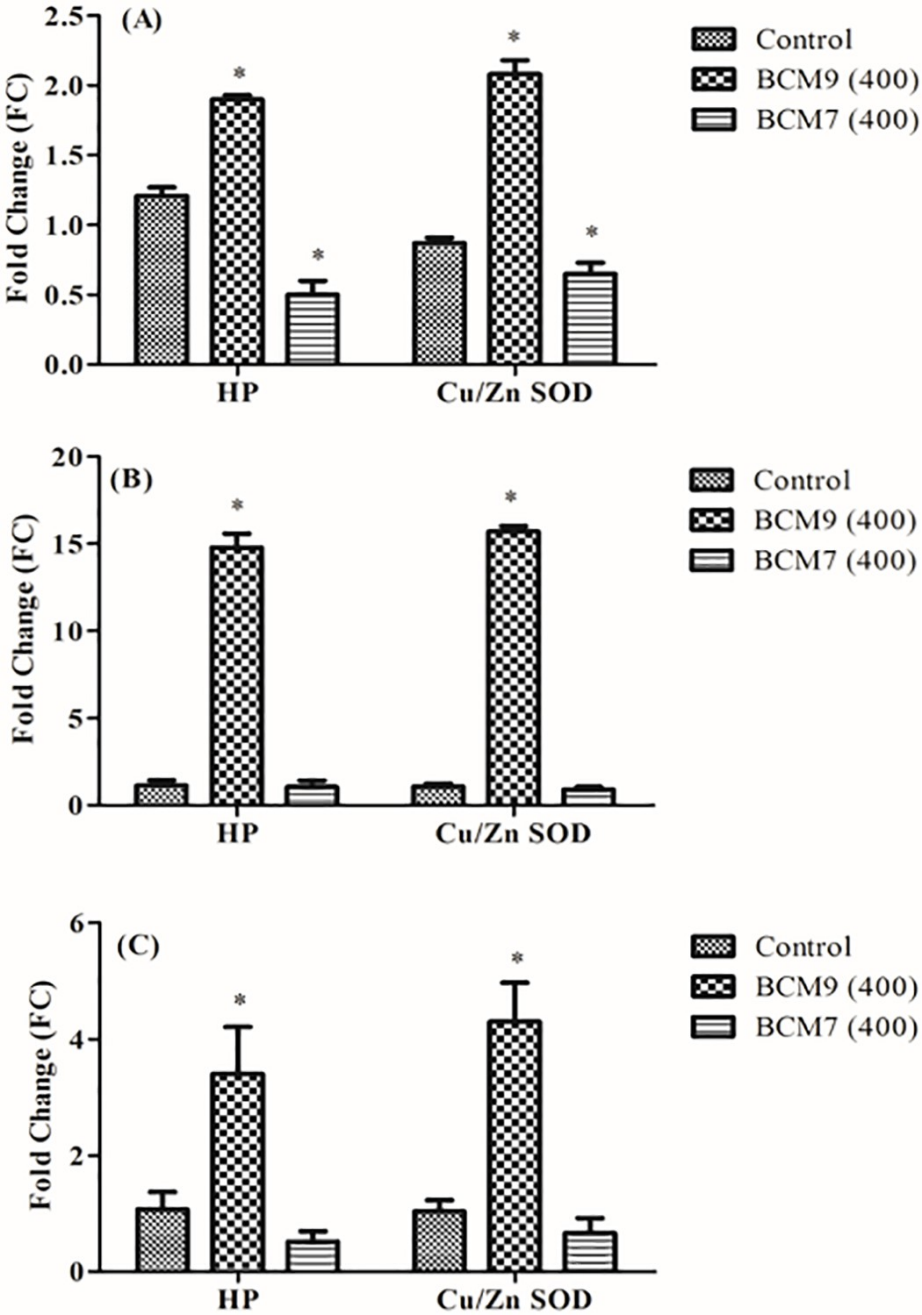
Relative expression pattern of *HP* and *Cu/Zn SOD* in mice liver tissue injected with BCM7 and BCM9 peptide after normalization with different internal control genes (A) *PPIA* and *SDHA* -the most stable (B) *TFR2* and *TBP* -the least stable and (C) *ACTB-* commonly used internal control gene. *Indicates significant difference (p<0.05) with reference to control group.

To validate the stability of the proposed best internal control genes, the expression profile of target genes reported to be associated with diabetes in mice liver was measured. Changes in expression of cytosol copper superoxide dismutase (*Cu/Zn SOD*) that convert superoxide into hydrogen peroxide [35] and acute phase anti-inflammatory protein coding haptoglobin (HP) [36] gene were analyzed using the most stable (*PPIA* and *SDHA*), least stable (*TFR2* and *TBP*) and one of the commonly used (*ACTB*) internal control gene for normalization of expression data (Fig 6). The relative expression of *HP* and *Cu/Zn SOD* increased in mice liver injected with BCM9 as compared to control (*HP*-1.6 and *Cu/Zn SOD*-2.4 fold) while in BCM7 treated mice, the expression levels were lower (*HP*-2.4 and *Cu/Zn SOD*-1.3 fold) compared to control when the normalization was performed using the most stable internal control genes (Fig 6A). In comparison, on normalization with the least stable internal control genes (*TFR2* and *TBP*), both *HP* (12.95 fold) and *Cu/Zn SOD* (14.67 fold) were found to be overexpressed nearly 14 times in liver tissue of BCM9 injected mice whereas the expression level in BCM7 injected mice was almost similar to that of control group (Fig 6B). Further, on normalization with *ACTB*, one of the commonly used internal control gene, the comparative pattern of gene expression for both *HP* and *Cu/Zn SOD* across the three groups was similar to that when normalization was performed with most stable internal control genes, however, the fold change increase in BCM9 (*HP*-3.16; *Cu/Zn SOD*-4.20 fold) group and decrease in BCM7 group (*HP*-2.06, *Cu/Zn SOD*-1.58 fold) compared to control was relatively higher (Fig 6C).

However, the fold change in expression level of both the candidates was higher in BCM9 (*HP*-3.16; *Cu/Zn SOD*-4.20 fold) group and lower in BCM7 group (*HP*-2.06, *Cu/Zn SOD*-1.58 fold) compared to control was relatively higher (Fig 6C).

## Discussion

Many studies have emphasized the importance of normalization of target gene expression data from qPCR analyses for its accurate and reliable quantitation. Validation of ICGs for different experimental conditions is essential as using ICGs with variable expression could lead to biased results and misinterpretation of the actual observations [37, 38]. Bustin *et al*. [39] have also defined MIQE guidelines for the selection of more than one internal control gene for normalization and accurate analysis of real-time PCR based gene expression data. Combination of different statistical algorithms is being employed to assess the suitability of ICGs, due to lack of consensus upon which approach is the best. In this study, raw Cq values obtained from qPCR were rigorously analyzed using nonparametric tests and statistical programs like geNorm, NormFinder and BestKeeper, so that only the most stably expressed genes are selected for data normalization. Amongst the three approaches, geNorm calculates the gene expression stability value (M-value) as the average pairwise variation of a particular gene with all other ICGs tested simultaneously. Herein, the gene with the highest M-value is excluded stepwise as M-value is negatively correlated to gene expression stability. NormFinder, a model-based approach ranks the set of candidate genes according to their expression stability calculated from linear scale expression transformed by delta-CT method, the lowest ranking represents the lowest variation and most stable expression. BestKeeper evaluates each candidate ICG in terms of its coefficient of correlation to an index (geometric mean of all candidate genes). It also calculates both the SD and coefficient of variation (CV) of the original Cq values and candidate genes showing the lowest calculated variations (CV ± SD) have the greatest stability.

Several authors have reported candidate reference genes, using geNorm, NormFinder and BestKeeper software in different cell types across different species like cattle [30], mice [28] and goat [40]. Identification of different internal control genes in various mice tissues like oocytes and embryos [41], skeletal muscle [27, 42], liver [28, 42–44], adipose tissue [42], brain [45], osteoblasts and macrophages [46], also indicate the suitability of these software in deciding the single or multiple genes for expression data normalization.

In our study we not only tried to establish the most suitable ICGs for liver of C57BL/6 mice administered with cow milk derived beta-casomorphins; BCM7 and BCM9, but also tested the variation in expression of individual ICGs between the groups. After analyzing the panel of ten candidate ICGs in this experiment, it was observed that *PPIA*, *ACTB*, *SDHA*, *B2M* and *HMBS* were the most stably expressed ICGs in liver tissue of the mice injected with BCM7/BCM9 peptides. The statistical programs, used to analyze the qPCR gene expression data, have also been recommended in many other studies for different species or cell types for the identification of the best suitable ICGs [28–30, 47, 48]. Day et al [28] revealed that the expression of different candidate ICGs changes even in different lobes of liver and variation in treatment type also affects the expression of different ICGs. The investigators concluded that *ACTB* and *Ywhaz* are the most suitable endogenous genes expressed in homogenous mice liver tissue after treatment with polyphenols. In another study on acute alcoholic liver injury response in mouse model, *Hprt1* and *Gapdh* has been reported as the best gene pair for qPCR data normalization in hepatic tissues using geNorm, NormFinder and BestKeeper tools [49]. Similarly, other reports also indicated that *ACTB* is not the stably expressed gene in mice liver that has undergone fibrosis due to schistosomiasis rather *Gapdh* showed relatively stable expression in infected as well as healthy mouse liver [21]. These results thus point towards the necessity of selection of suitable genes for normalizing gene expression data with each experiment, as confirmed by our results as well. In the present study, we observed that *TFR2* and *TBP* genes were least stably expressed throughout the experiment although from BestKeeper analysis, *TBP* gene had lower SD value, but in BestKeeper vs. reference gene index, based on the coefficient of correlation (r) its value was lower in comparison to other candidate genes. Though, there was variation in the ranking of different reference genes using three algorithms, *PPIA* was identified as most stably expressed ICG by geNorm as well as NormFinder and also the SD value was <1 by BestKeeper.

Though, best reference genes for a cell type/tissue/organ cannot be generalized for all experimental conditions. *PPIA* having remarkable evolutionary conservation and broad cellular and tissular distribution, has been observed as one of the most stable genes expressed in mice liver tissue. Gong *et al*. [42] reported *PPIA* and *B2M* being the most stable genes expressed in mice liver tissue during caloric restriction experiments using the same three statistical tools as that of the present study. *PPIA* has also been reported by Takagi *et al*. [50], as one of the most stable genes in direct hyperplasia of liver in female C57BL/6 mice using geNorm and NormFinder. In case of liver regeneration after any injury, again the expression of *PPIA* gene was found to be the most stable [44]. Previous studies, also supported by present findings, thus indicate expression of *PPIA* gene is least affected under different pathophysiological conditions of the mouse liver and could be utilized during different experiments on mice liver. However, validation of same for a specific experimental set up is still required.

Suitability of the identified internal control genes (ICGs) was checked by assessing the relative expression pattern of *HP* and *Cu/Zn SOD* across control and treatment (BCM9 and BCM7) groups upon normalization with most stable (*PPIA* and *SDHA*), least (*TFR2* and *TBP*) stable ICGs identified in the present study and one of the commonly used ICG (*ACTB*) in expression analysis. The selected target genes (*HP* and *Cu/Zn SOD*) have a defensive role against diabetes as their increased expression helps to prevent diabetes mellitus by decreasing oxidative stress. On the contrary, comparatively lower expression has been observed for these target genes in diabetic tissues. Expression levels of *HP* and *Cu/Zn SOD* normalized with the best ICG combination were significantly different from those normalized with the least stable ICG (Fig 6A & 6B). With the best ICGs for normalization, compared to control group, there was nearly 2 fold increase in the expression of *HP* (1.6 fold) *and Cu/Zn SOD* (2.4 fold) in mice liver tissue injected with BCM9 peptide while decrease in expression level of both the target genes (*HP*-2.4 fold *and Cu/Zn SOD* 1.3 fold) was observed in mice group injected with BCM7. These changes in the relative expression profile are in line with that of earlier reports [50, 51]. Upon normalization with the least stable ICG, an unexpected higher expression was observed both for *HP* (12.95 fold) and *Cu/Zn SOD* (14.67 fold) in BCM9 group compared to control and BCM7 group. Conversely, there was no significant difference in the expression pattern of these genes in control *vs* BCM7 group. Further, upon normalization of expression data with *ACTB*, one of the commonly used internal control gene in such studies, though, the expression levels were comparatively higher in BCM9 group than expected, pattern of relative changes in candidate gene expression profile (BCM9>control>BCM7 group) was similar to one observed when most stable ICGs were used for normalization (Fig 6C). This might be attributed to the fact that in our study, ACTB is ranked among the top five stable genes. The use of different ICGs could objectively reflect the changes in expression levels of target genes and hence validate the reliability of the identified stable internal control genes.

To the best of our knowledge, this is the first report on identification of internal control genes in liver tissue of mice injected with BCM peptides. The results clearly establish that there is a need to use all the three software for the selection of most stably expressed genes and *PPIA*, *ACTB*, *B2M*, *SDHA* and *HMBS* genes can be used in combination for the best normalization of target genes expression for the liver of BCM7/9 peptides treated mice.

## Supporting information

S1 Fig Gel image representing amplified product for different genes. Lanes left to right: Lane1-*ACTB*, lane2*-TBP*, lane3*-PGK1*, lane4*-B2M*, lane5*-PPIA*, lane6*-GUSB*, lane7*-HPRT*, lane8*-HMBS*, lane9*-TFR2* and lane10*-SDHA* gene *and* M-100 bp marker (New England Biolabs, N3231).
(DOCX)

S2 Fig Melting curve analysis of all 10 candidate ICGs. (DOCX)

S3 Fig The data of expression levels of individual candidate ICGs across control and treatment groups staged as quantification cycle (Cq) values of each gene in the box-whisker diagram. (DOCX)

S4 Fig Relative quantity of HP and Cu/Zn SOD in mice liver tissue injected with BCM7 and BCM9 peptide after normalization with ACTB: commonly used ICG (A) and PPIA: most stable ICG (B). (DOCX)

S1 Table 260/230 and 260/280 ratios for RNA isolated from different biological samples. (DOCX)

S1 Data (RAR)
