# Supplementary material for: Identification of stably expressed Internal Control Genes (ICGs) for normalization of expression data in liver of C57BL/6 mice injected with beta casomorphins
Source: PLoS One. 2023 May 5;18(5):e0282994. doi: 10.1371/journal.pone.0282994 (PMC10162558; doi:10.1371/journal.pone.0282994)
Supplement: S1 Fig — Lanes left to right: Lane1-ACTB, lane2-TBP, lane3-PGK1, lane4-B2M, lane5-PPIA, lane6-GUSB, lane7-HPRT, lane8-HMBS, lane9-TFR2 and lane10-SDHA gene and M-100 bp marker (New England Biolabs, N3231). (DOCX) [file pone.0282994.s001.docx]

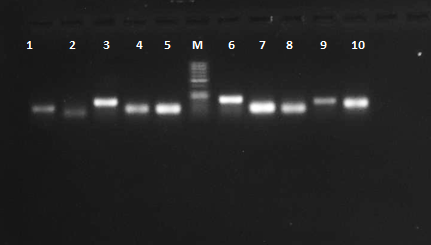


**S1 Fig. Gel image representing amplified product for different genes. Lanes left to right: Lane1-*ACTB,* lane2*-TBP,* lane3*-PGK1,* lane4*-B2M,* lane5*-PPIA,* lane6*-GUSB,* lane7*-HPRT,* lane8*-HMBS,* lane9*-TFR2* and lane10*-SDHA* gene *and* M-100 bp marker** (New England Biolabs, N3231)**.**
