# Supplementary material for: Identification of stably expressed Internal Control Genes (ICGs) for normalization of expression data in liver of C57BL/6 mice injected with beta casomorphins
Source: PLoS One. 2023 May 5;18(5):e0282994. doi: 10.1371/journal.pone.0282994 (PMC10162558; doi:10.1371/journal.pone.0282994)
Supplement: S2 Fig — (DOCX) [file pone.0282994.s002.docx]

**S2 Fig. Melting curve analysis of all 10 candidate ICGs.**
