# Supplementary material for: Identification of stably expressed Internal Control Genes (ICGs) for normalization of expression data in liver of C57BL/6 mice injected with beta casomorphins
Source: PLoS One. 2023 May 5;18(5):e0282994. doi: 10.1371/journal.pone.0282994 (PMC10162558; doi:10.1371/journal.pone.0282994)
Supplement: S3 Fig — (DOCX) [file pone.0282994.s003.docx]

**
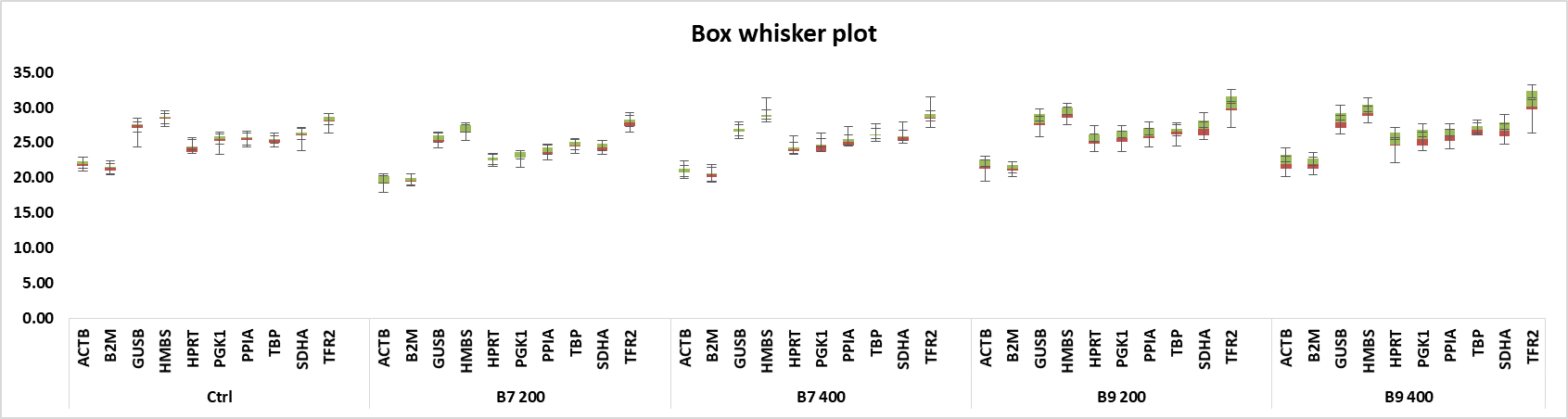
**

**S3 Fig. The data of expression levels of individual candidate ICGs across control and treatment groups staged as quantification cycle (Cq) values of each gene in the box-whisker diagram.**
