# Supplementary material for: Identification of stably expressed Internal Control Genes (ICGs) for normalization of expression data in liver of C57BL/6 mice injected with beta casomorphins
Source: PLoS One. 2023 May 5;18(5):e0282994. doi: 10.1371/journal.pone.0282994 (PMC10162558; doi:10.1371/journal.pone.0282994)
Supplement: S4 Fig — (DOCX) [file pone.0282994.s004.docx]

**
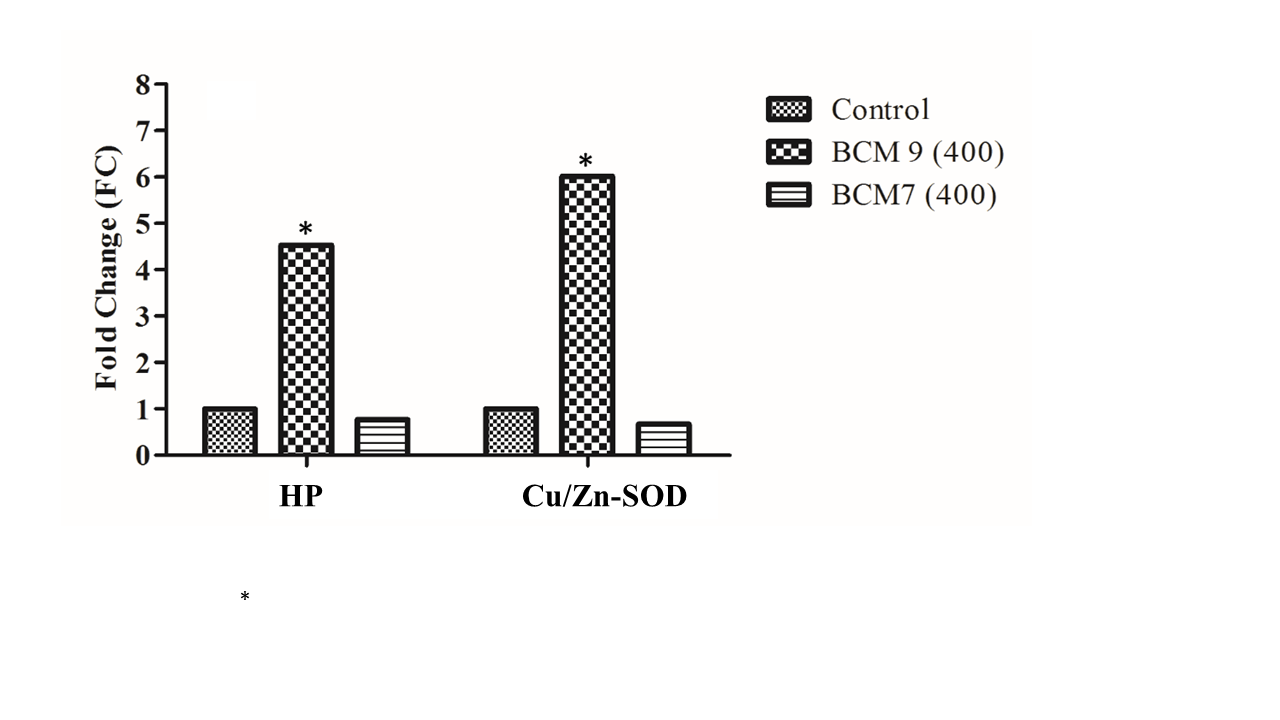
**

**S4 Fig. Relative quantity of *HP* and *Cu/Zn SOD* in mice liver tissue injected with BCM7 and BCM9 peptide after normalization with commonly used ICG (*ACTB*)**
