# Supplementary material for: Identification of stably expressed Internal Control Genes (ICGs) for normalization of expression data in liver of C57BL/6 mice injected with beta casomorphins
Source: PLoS One. 2023 May 5;18(5):e0282994. doi: 10.1371/journal.pone.0282994 (PMC10162558; doi:10.1371/journal.pone.0282994)
Supplement: S1 Table — (DOCX) [file pone.0282994.s005.docx]

**S1 Table. 260/230 and 260/280 ratios for RNA isolated from different biological samples**

| **Groups** | **Sample No.** | **A260/280** | **A260/230** |
| --- | --- | --- | --- |
| Control-  Sample No. (1-6) | 1 | 2.06 | 2.12 |
|  | 2 | 2 | 2.08 |
|  | 3 | 1.97 | 2.01 |
|  | 4 | 2.07 | 2.11 |
|  | 5 | 2.08 | 2.2 |
|  | 6 | 2.08 | 2.13 |
| BCM7:200 µg group - Sample No. (7-13) | 7 | 2.09 | 2.2 |
|  | 8 | 2.07 | 2.12 |
|  | 9 | 2.05 | 2.1 |
|  | 10 | 2.08 | 2.0 |
|  | 11 | 2.07 | 2.1 |
|  | 12 | 2.07 | 2.17 |
|  | 13 | 2.09 | 2.12 |
| BCM7:400 µg group - Sample No. (14-18) | 14 | 2.09 | 2.21 |
|  | 15 | 1.94 | 2.15 |
|  | 16 | 2.01 | 2.1 |
|  | 17 | 2.05 | 2.15 |
|  | 18 | 2.00 | 2.07 |
| BCM9:200 µg group - Sample No. (19=24) | 19 | 2.03 | 2.09 |
|  | 20 | 2.04 | 2.16 |
|  | 21 | 2.05 | 2.12 |
|  | 22 | 2.06 | 2.2 |
|  | 23 | 2.05 | 2.08 |
|  | 24 | 2.08 | 2.1 |
| BCM9:400 µg group - Sample No. (25-30) | 25 | 2.08 | 2.2 |
|  | 26 | 2.05 | 2.11 |
|  | 27 | 2.07 | 2.21 |
|  | 28 | 2.08 | 2.1 |
|  | 29 | 2.02 | 2.13 |
|  | 30 | 1.99 | 2.09 |
| Mean± SD |  | 2.05 ± 0.04 | 2.12 ± 0.05 |
